# Relaxed fibronectin: a potential novel target for imaging endometriotic lesions

**DOI:** 10.1186/s13550-024-01070-0

**Published:** 2024-02-10

**Authors:** Belinda Trachsel, Stefan Imobersteg, Giulia Valpreda, Gad Singer, Regula Grabherr, Mark Ormos, Irene A. Burger, Rahel A. Kubik-Huch, Roger Schibli, Viola Vogel, Martin Béhé

**Affiliations:** 1 Center for Radiopharmaceutical Sciences ETH-PSI-USZ, Paul Scherrer Institute, Forschungsstrasse 111, 5232 Villigen, Switzerland; 2 Department of Chemistry and Applied Biosciences, Institute of Pharmaceutical Sciences, ETH Zurich, 8093 Zurich, Switzerland; 3 Laboratory of Applied Mechanobiology, Institute of Translational Medicine, Department of Health Sciences and Technology, ETH Zurich, 8093 Zurich, Switzerland; 4 Kantonsspital Baden, 5404 Baden, Switzerland

**Keywords:** Endometriosis, Fibronectin, FnBPA5, Extracellular matrix, Radiopharmacy, Estrous cycle

## Abstract

**Background:**

Endometriosis is characterized by the ectopic occurrence of endometrial tissue. Though considered benign, endometriotic lesions possess tumor-like properties such as tissue invasion and remodeling of the extracellular matrix. One major clinical hurdle concerning endometriosis is its diagnosis. The diagnostic modalities ultrasound and MRI are often unable to detect all lesions, and a clear correlation between imaging and clinical symptoms is still controversial. Therefore, it was our aim to identify a potential target to image active endometriotic lesions.

**Results:**

For our studies, we employed the preclinical radiotracer [^111^In]In-FnBPA5, which specifically binds to relaxed fibronectin–an extracellular matrix protein with key functions in homeostasis that has been implicated in the pathogenesis of diseases such as cancer and fibrosis. We employed this tracer in biodistribution as well as SPECT/CT studies in mice and conducted immunohistochemical stainings on mouse uterine tissue as well as on patient-derived endometriosis tissue. In biodistribution and SPECT/CT studies using the radiotracer [^111^In]In-FnBPA5, we found that radiotracer uptake in the myometrium varies with the estrous cycle of the mouse, leading to higher uptake of [^111^In]In-FnBPA5 during estrogen-dependent phases, which indicates an increased abundance of relaxed fibronectin when estrogen levels are high. Finally, immunohistochemical analysis of patient samples demonstrated that there is preferential relaxation of fibronectin in the proximity of the endometriotic stroma.

**Conclusion:**

Estrous cycle stages characterized by high estrogen levels result in a higher abundance of relaxed fibronectin in the murine myometrium. This finding together with a first proof-of-concept study employing human endometriosis tissues suggests that relaxed fibronectin could be a potential target for the development of a diagnostic radiotracer targeting endometriotic lesions. With [^111^In]In-FnBPA5, the matching targeting molecule is in preclinical development.

**Supplementary Information:**

The online version contains supplementary material available at 10.1186/s13550-024-01070-0.

## Introduction

Endometrial tissue eloping the uterine cavity and residing in distant organs such as the peritoneum or the ovaries is termed endometriosis. It is a highly prevalent disease among women of childbearing age, with an estimated 10% suffering from this debilitating condition [1]. Though endometriosis is considered benign, it leads to a drastic reduction in the quality of life with manifold symptoms including heavy menstrual bleeding and accompanying pain, symptoms of the digestive tract as well as reduced fertility [2] and increased risk of ovarian cancer [3]. The etiology of endometriosis remains an enigma. However, the most widely accepted theory is retrograde menstruation, a process in which endometrial cells elope from the uterine cavity through the fallopian tubes during menstruation, finding ectopic adhesion sites in the peritoneal cavity or in other distant tissues [4].

A crucial role in both pathogenesis and progression of endometriosis is attributed to the extracellular matrix (ECM). In healthy tissues, this network of proteins and soluble factors provides a scaffold for cells and is highly involved in many physiological processes such as cell–cell communication, proliferation and differentiation. Being a highly dynamic space, the ECM is readily degraded and built up again according to the needs of the corresponding tissue. In states of disease, this balance is in disequilibrium and the ECM undergoes changes in composition, architecture and strain. In the pathogenesis of endometriosis, the ECM is involved at an early stage by establishing a pro-endometriotic niche that allows endometrial stromal cells to adhere and establish vascularization [5].

However, the ECM is not only involved in the establishment of endometriosis, there is overwhelming evidence that endometriotic tissue undergoes extensive matrix remodeling. A key factor in this process are metalloproteinases (MMPs), which readily degrade many matrix proteins during physiological processes such as cell proliferation and differentiation. In patients suffering from endometriosis, MMPs are increased in serum [6] and peritoneal fluid [7] and are overexpressed in ectopic and/or eutopic endometrium compared with healthy women [8–10]. This excess in MMPs results in uncontrolled matrix degradation and is aggravated by a decrease in tissue inhibitors of metalloproteinases (TIMPs) [7], which normally regulate tissue degradation by inhibiting MMP activity, creating a tightly regulated equilibrium essential for physiological processes.

Likewise, there is now substantial evidence that fibronectin (Fn) is involved in the pathogenesis of endometriosis. Fn is a dimeric ECM protein composed of two 230–250 kDa subunits connected at the C-termini through disulfide bridges [11]. It is vital for life [12] and is an integral part of the ECM with important functions in hemostasis and wound healing. Besides interacting with other molecules, Fn proteins also readily polymerize with each other, forming large fibrils [13], which through mechanical stretching regulate molecular and cellular functions [14].

With respect to endometriosis, a large meta-analysis combining 11 genome-wide association studies demonstrated that the FN1 gene, encoding Fn, is associated with moderate to severe endometriosis [15]. Other studies showed allelic and genetic differences [16–18] in FN1 between patients and healthy subjects and increased expression of the splice variant Fn-EDB in endometriotic lesions compared with eutopic endometrium [19]. On the protein level, the findings remain controversial. While Béliard et al. found no difference in Fn expression between the eutopic and ectopic endometrium [20], Holzer et al. found decreased Fn expression in the eutopic endometrium [18] and Zhang et al. reported increased Fn expression in the ectopic endometrium [21]. Despite the apparent controversy of Fn expression in eutopic and ectopic endometrium, Fn levels in plasma [22, 23] and peritoneal fluid [23] from endometriosis patients are consistently reported as elevated compared to healthy women.

A specific imaging probe would be highly sought-after as current diagnostic imaging approaches are unsatisfactory, resulting in an average of 7 years until confirmation of diagnosis [24]. The standard methods used in the clinics for imaging of endometriotic lesions to date are transvaginal ultrasound (TVUS) and magnetic resonance imaging (MRI). Both have their respective shortcomings; ultrasound can only visualize lesions in the proximity of the uterus and MRI is highly sensitive in detecting blood remnants and actively bleeding lesions, but loses sensitivity in fibrotic lesions and small bowel involvement and requires time-intense analysis and female imaging expertise by radiologists. Importantly, none of them can distinguish between symptomatic and asymptomatic endometriosis [25]. The gold standard of endometriosis diagnosis therefore remains pathological assessment following laparoscopy.

For improved treatment regimens and better surgical planning prior to laparoscopy, localization of endometriosis sites and extent would be crucial. As conventional imaging methods fail to meet these requirements, molecular imaging, e.g., in the form of a novel positron emission tomography (PET) radiotracer that specifically targets endometriotic lesions, could close this gap. In fact, several targets and targeting moieties have already been explored: [^18^F]FDG was reported to show uptake in endometriotic lesions, [26–28] but with a high false-positive rate [29, 30]. Other approaches were directed toward targeting of the somatostatin receptor [31], the estrogen receptor [32, 33] or the nociceptive receptor sigma-1 [34]. However, to date no radiotracer is admitted for clinical use in the diagnosis of endometriosis.

**Fig. 1:**
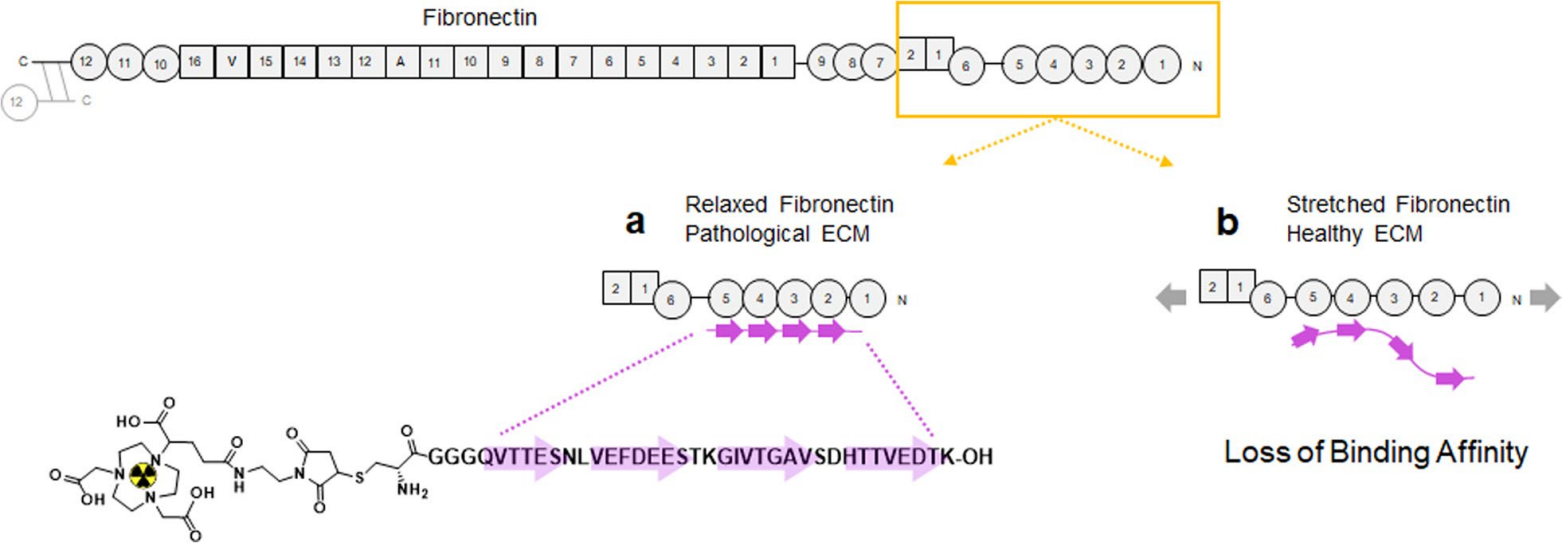
Schematic overview of the binding of [^111^In]In-FnBPA5 to fibronectin (Fn) depending on the mechanical conformation. **a** In the pathological ECM a high abundance of relaxed Fn can be detected. If Fn fibers are relaxed, [^111^In]In-FnBPA5 binds with nM affinity to Fn. **b** In healthy tissues Fn fibers are predominantly stretched. Due to structural mismatch [^111^In]In-FnBPA5 loses affinity to stretched Fn. Figure components adapted from Arnoldini et al. [35] CC by 4.0

In our laboratory, we have previously developed the mechanosensitive peptide FnBPA5 by radiolabeling with the ɣ-emitter indium-111 into the radiotracer [^111^In]In-FnBPA5 [35]. [^111^In]In-FnBPA5 consists of an amino acid sequence naturally expressed in the pili of a wide-range of bacteria, which through high affinity binding to the N-terminal region of relaxed Fn (Fig. 1), allows them to infect wounds and provides motility within the host tissue. However, due to structural mismatch, FnBPA5 loses most of its affinity toward stretched Fn fibers [36, 37]. Fn fibers under pathological conditions such as in the cancer-associated ECM are preferentially relaxed, which results from a combination of processes such as increased expression of Fn, loss of cell-mediated pulling, increased cross-linking and faster proteolytic degradation [38]. Based on the increased expression of MMPs, the profound matrix remodeling in ectopic endometrium as well as based on our serendipitous finding that [^111^In]In-FnBPA5 accumulates in the uterus of mice, we hypothesize that [^111^In]In-FnBPA5 could serve as a novel imaging probe to detect and localize endometriotic lesions.

## Results

### [^111^In]In-FnBPA5 accumulates in the mouse uterus with up to 17% iA/g

While visualizing the radiotracer [^111^In]In-FnBPA5 in female tumor-bearing mice using SPECT/CT, we detected an unanticipated radiotracer signal in the lower abdomen of some but not all animals. Closer investigation of this phenomenon through quantification of radioactivity within single organs by means of a biodistribution study verified that [^111^In]In-FnBPA5 accumulates in the mouse uterus (see Additional file 1: Table S1). Interestingly, this uptake varied tremendously between mice from 0.7 to 17% iA/g. Intrigued by this finding, we speculated that the hormonal variation induced by the murine estrous cycle could affect the expression of relaxed Fn in the uterus. To prove our hypothesis, we continued our investigation by correlating the uptake of [^111^In]In-FnBPA5 to the estrous cycle stage.

### Differential accumulation in the uterus is mediated by the estrous cycle

**Fig. 2:**
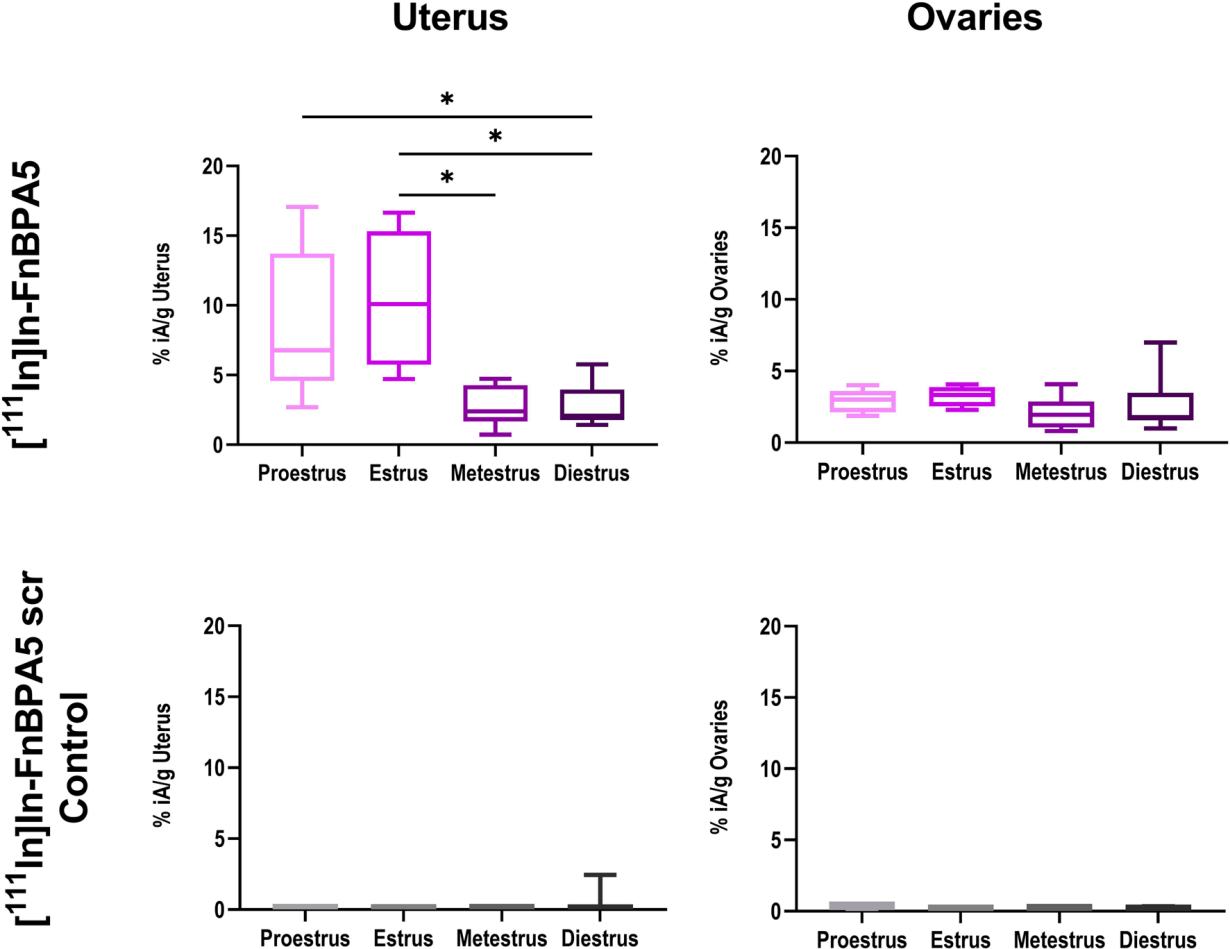
Accumulation of [^111^In]In-FnBPA5 and the control peptide [^111^In]In-FnBPA5 scr in the uterus and the ovaries according to the estrous cycle stage of the mouse 24 h after i.v. injection. While the uterus accumulation of [^111^In]In-FnBPA5 is mediated by the estrous cycle, with increased uptake during proestrus and estrus, uptake of [^111^In]In-FnBPA5 in the ovaries is unaffected by hormonal changes. In contrast to [^111^In]In-FnBPA5, the scrambled control [^111^In]In-FnBPA5 scr displays only background uptake in both uterus and ovaries independent of the estrous cycle stage of the mouse. Statistical significance was assessed using Kruskal–Wallis and Dunn’s post hoc test with *p < 0.05. *n* = 6 (proestrus), 4 (estrus), 7 (metestrus), 12 (diestrus) for [^111^In]In-FnBPA5 and *n* = 3 (proestrus), 2 (estrus), 5 (metestrus), 10 (diestrus) for [^111^In]In-FnBPA5 scr

Based on our hypothesis, we defined the estrous cycle stage (proestrus, estrus, metestrus and diestrus) of each mouse by microscopic analysis of vaginal smears (Additional file 1: Figure S2) and correlated it with the radiosignal of [^111^In]In-FnBPA5 in the uterus. Figure 2 depicts the differential tracer uptake in the uterus as a function of the estrous cycle stage of the mouse at 24 h p.i. While progesterone-dependent stages diestrus and metestrus display low uterine uptake of 2.7 ± 1.4% iA/g and 2.6 ± 1.4% iA/g, both proestrus and estrus lead to significantly higher tracer accumulation in the uterus of 8.7 ± 5.4% iA/g and 10.4 ± 5.0% iA/g, respectively. A similar effect was not observed for any other organ, not even for the ovaries, which are also highly influenced by hormonal changes.

Apart from remodeling of the matrix, muscular contractions and increased perfusion induced by changes in estrogen and progesterone levels can affect the radiotracer distribution. Therefore, to confirm the specificity of the observed effect, a scrambled version of FnBPA5 termed FnBPA5 scr (GSEQEDLTGTKVDFGETIVVNEATETVTSGSTHGTKV), which does not bind to Fn, was employed as a control peptide. Biodistribution of [^111^In]In-FnBPA5 scr confirmed that the uterine accumulation of [^111^In]In-FnBPA5 is specific to relaxed Fn and that other estrous cycle-mediated processes, especially increased perfusion, do not promote unspecific tracer uptake in the uterus (Fig. 2).

**Fig. 3:**
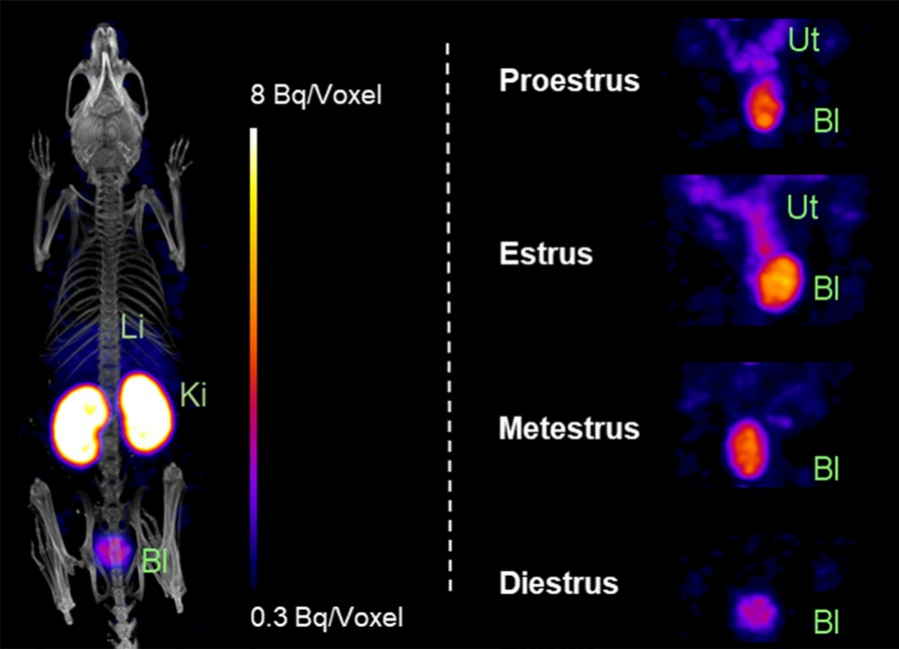
SPECT/CT images of mice in proestrus, estrus, metestrus and diestrus cycle stage 24 h after i.v. injection of ~15 MBq of [^111^In]In-FnBPA5. While there is a high activity in kidney and bladder associated with the elimination of the peptidic radiotracer, both mice in proestrus and estrus display uptake in the uterus. Li = liver, Ki = kidney, Bl = bladder

Using SPECT/CT, the differential uptake of [^111^In]In-FnBPA5 in the uterus at 24 h p.i. can be visualized (Fig. 3). The main elimination routes of peptidic radiotracers are the kidneys and the bladder, leading to high unspecific uptake of [^111^In]In-FnBPA5 in these organs. In addition to the kidneys and the bladder, animals in the proestrus and estrus cycle stages show profound radiotracer uptake in the typical shape of the uterus located between the kidneys and the bladder.

### Changes in the myometrium of the mouse induce relaxation of fibronectin

**Fig. 4:**
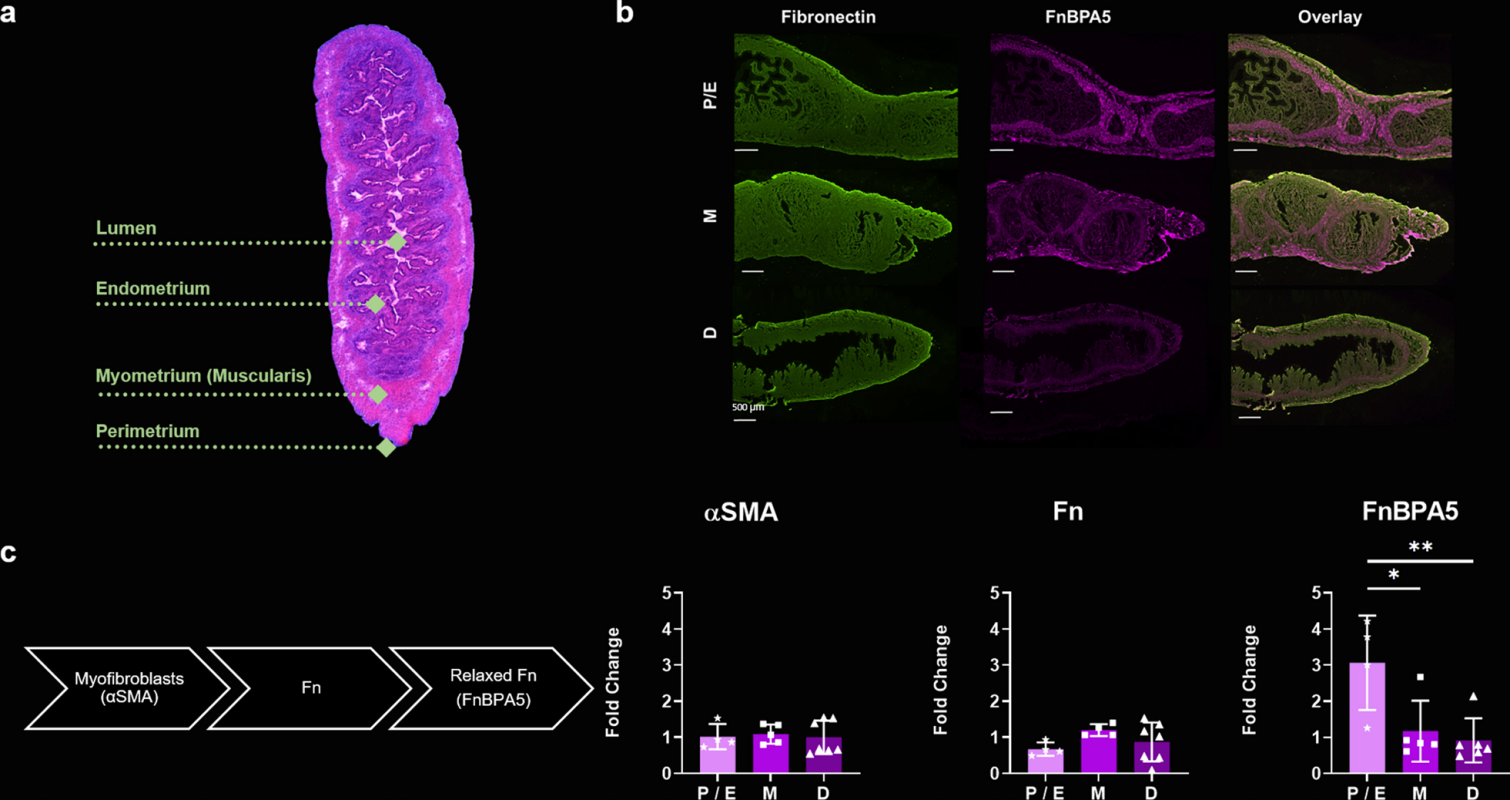
**a** Histological view of a mouse uterus stained with H&E depicting the different layers making up the uterine wall. **b** Immunohistochemical stains of mouse uteri in proestrus/estrus (P/E), metestrus (M) or diestrus (D) stained for Fn (green) and relaxed Fn using FnBPA5 (pink). While there is a constant and homogenous expression of Fn, there is a distinctly higher abundance of relaxed Fn (FnBPA5) in the myometrium during proestrus/estrus (P/E). All images are shown in Additional file 1: Figures S4a-c. **c** Quantitative analysis of the fluorescent signal in the myometrium of uteri in different cycle stages. αSMA is a marker for myofibroblasts, which are the main producers of Fn in tissues. While there is no change in αSMA and Fn signal in the myometrium along the estrous cycle, relaxed Fn (FnBPA5) is significantly more abundant during proestrus/estrus. Statistical analysis was conducted using one-way ANOVA with Tukey’s multiple comparison post hoc test (*p < 0.05, **p < 0.01) Raw data is summarized in Additional file 1: Table S3

To investigate our observation of increased radiotracer accumulation in the uterus on the tissue level, we performed immunohistochemistry with uterus tissue from different estrous cycle stages. Figure 4a shows the morphology of a mouse uterus visualized using hematoxylin & eosin (H&E), with the luminal endometrium, the muscular myometrium and the surrounding perimetrium. Immunohistochemical staining for Fn and relaxed Fn (using Cy5-FnBPA5) shows that while Fn expression is largely homogenous and unaffected by hormonal changes, there is a pronounced increase in the amount of relaxed Fn, which is distinctly located in the myometrium during the estrogen-dependent phases (proestrus/estrus) (Fig. 4b). Delving deeper into the myometrium, we quantitatively analyzed the density of myofibroblasts—the major producers of cFn–in the myometrium using αSMA as a marker and correlated it with the respective abundance of Fn and relaxed Fn (Fig. 4c). While the myometrium shows constant expression of αSMA and Fn regardless of the estrous cycle stage, relaxed Fn is almost threefold more abundant in the myometrium in proestrus/estrus than in metestrus or diestrus. Coherently, the higher abundance of relaxed Fn is not caused by increased Fn expression, but must be related to breakdown mechanisms or cellular processes that cause tissue relaxation.

### FnBPA5 Co-localizes with endometriotic stroma, but not with endometriotic epithelium

**Fig. 5:**
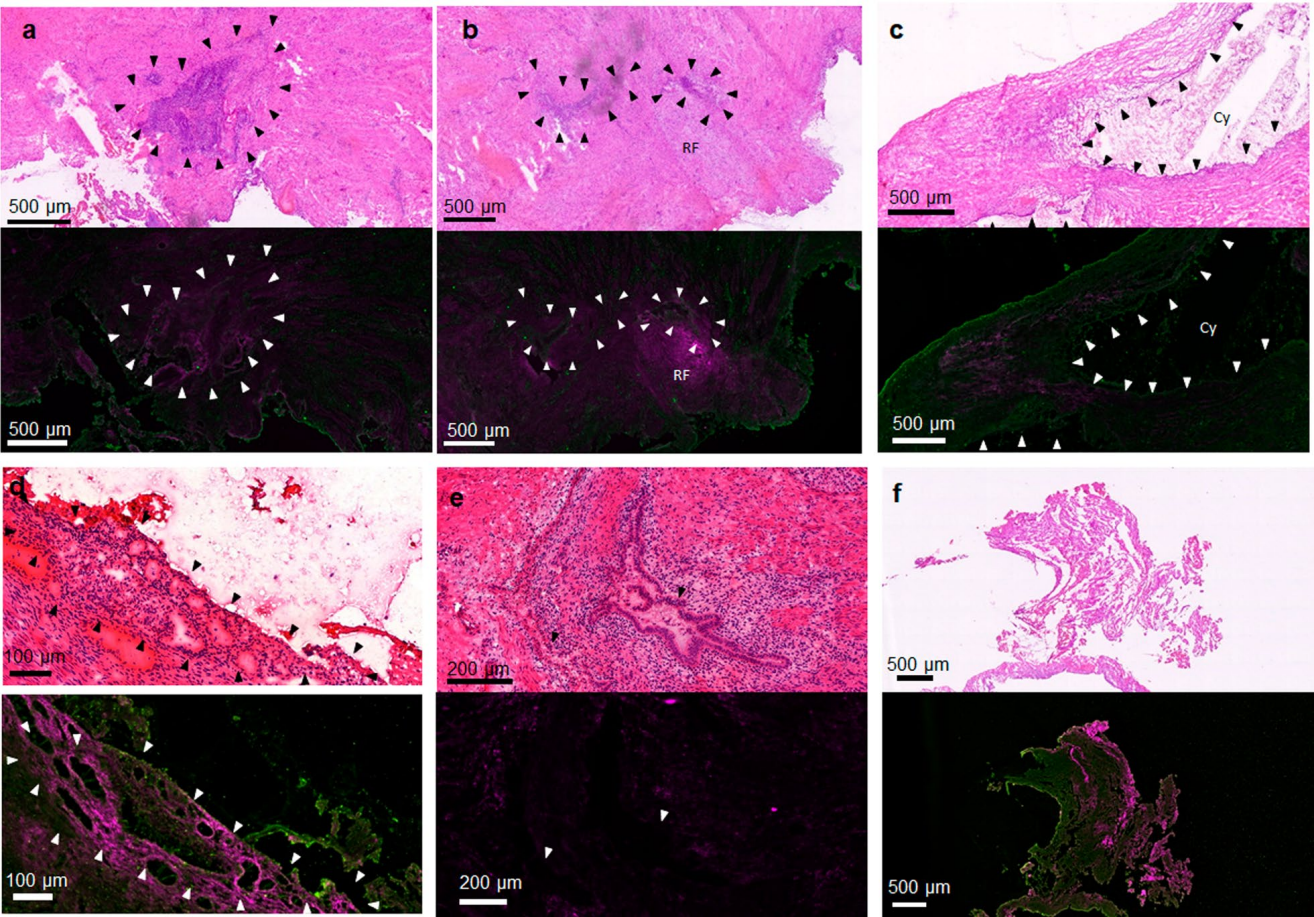
Examples of endometriotic lesions identified using H&E (top) and the corresponding areas stained with fluorescent markers. Arrowheads identify endometriotic tissue regions. Cell nuclei are stained with DAPI (blue), Fn is depicted in green and relaxed Fn (Cy5-FnBPA5) in pink. **a** An endometriotic lesion containing endometriosis stroma and epithelium with characteristic Cy5-FnBPA5 staining. **b** Same specimen as in (A), but another endometriotic region. Particularly, strong Cy5-FnBPA5 staining is observed in a region displaying reactive fibrosis (RF). **c** Disrupted cyst (Cy) lined by endometriotic epithelium. FnBPA5 shows strong signal in the proximity of the cyst, but spares the direct border of the cyst, which is lined by endometriotic epithelium. **d** A region of endometriotic stroma that displays especially strong staining for relaxed Fn.** e** Close-up on a characteristic endometriotic gland lined by endometriotic epithelium both by H&E and using Cy5-FnBPA5. Cy5-FnBPA5 is not staining the epithelium. **f** FnBPA5 staining in regions of thermal ablation caused as an artifact from surgical removal

Pathological analysis of a first cohort of endometriotic tissues samples from laparoscopy showed strong spatial correlation of Cy5-FnBPA5 staining with endometriotic lesions (Fig. 5a–d). While endometriotic epithelium, which is relatively deprived of extracellular components, remained unstained (Fig. 5e), Cy5-FnBPA5 mostly demarcated areas rich in endometriotic stroma. Interestingly, not all areas identified as endometriotic stroma upon histological assessment using hematoxylin & eosin (H&E) demonstrated Cy5-FnBPA5 accumulation, suggesting that apart from the relatively high abundance of matrix proteins in endometriotic stroma, other effects such as scar formation or inflammation play a role in mediating the relaxation of Fn. This is further supported by the fact that apart from endometriotic stroma, Cy5-FnBPA5 was located in a region of reactive fibrosis, which often results from aberrant ectopic endometrium.

## Discussion

While Fn in healthy tissues usually appears stretched, pathological tissues and tissues undergoing ECM remodeling often display an increase in relaxed Fn [35, 39]. In mice, we observed such an effect also in the uterus, where relaxed Fn was significantly enriched during the estrogen-dependent cycle stages proestrus and estrus.

Indeed, a relation between Fn and the female sex hormones progesterone and estrogen has been implied previously, with estrogen or withdrawal of progesterone described as stimulators of Fn expression both in healthy and disease environments [40–43]. However, all studies available to date are investigating the relation between Fn and female sex hormone levels, without differential analysis of the mechanical conformation of Fn. Therefore, our study is the first account of an association between estrogen and relaxed Fn.

Intriguingly, the higher abundance of relaxed Fn at high estrogen levels was located in the muscular myometrium, whereas remodeling of the ECM is mainly associated with the endometrium. In women, the endometrium is shed together with blood during menstruation only to be built up again during the next cycle. This shedding is accompanied by strong remodeling of the matrix [44]. In mice, however, except for the spiny mouse [45], the endometrium is broken down, partly recycled or shed without blood, but no menstruation occurs. Our findings therefore suggest that a similar matrix remodeling effect as a function of sex hormone levels occurs in the muscular component of the mouse uterus. Interestingly, relaxation of Fn is also observed in smooth muscle in the form of fibers or lining blood vessels in human endometriosis specimens, implying that this finding translates well from mouse to human. However, the exact mechanism and function behind this process remain unknown.

Despite the unknown nature of FnBPA5 accumulation in the murine myometrium, we hypothesized based on the detected estrous cycle dependent accumulation of FnBPA5 in the mouse uterus, that relaxed Fn could be a targeting vector for the localization of endometriotic lesions in women. Apart from the visualization of uterine tissue in mice, there are further reasons to believe that relaxed Fn could be a potential target for endometriosis: Firstly, literature findings have extensively shown aberrant expression of integrins in ectopic endometrium [20, 46–49]. As integrins are among the main binding partners of Fn, their differential expression has direct implications on the mechanical strain of Fn [50]. Secondly, increased estrogen levels (corresponding to proestrus and estrus in the mouse) are reported in endometriotic lesions [51, 52]. Thirdly, as described in the introduction, endometriotic lesions display increased MMP activity. In fact, MMPs are also regulated by estrogen [53–55] and, as described previously, are the main breakdown enzymes of Fn, potentially mediating its relaxation.

In a first proof-of-concept study, we therefore investigated the co-localization of FnBPA5 and endometriosis in 12 patients who had undergone laparoscopy and found a correlation between FnBPA5 staining and endometriotic stroma, but not with endometriotic epithelium. This locally increased presence of relaxed Fn in the stromal components of endometriosis is easily explained by the wealth of matrix in stroma and the absence thereof in epithelial tissue.

Though these first results from human biopsies are encouraging, further studies will need to assess the specificity of FnBPA5 toward endometriosis. In this regard, it will be key to elucidate whether FnBPA5 is able to distinguish eutopic endometrium and fibrotic scar tissue from ectopic endometrium and whether it could even differentiate between symptomatic and asymptomatic lesions. Finally, it will be up to the pharmacokinetic behavior of FnBPA5 in an in vivo setting to determine whether and to what extent FnBPA5 will accumulate in endometriotic lesions.

In general, the potential use of radiotracers in women of childbearing age should be discussed among nuclear physicians and endometriosis specialists, tightly weighing the risk and benefits of such a procedure. For a potential clinical application, we would also suggest the use of gallium-68, a positron emitter that is frequently used in the clinics instead of indium-111. Though we employed indium-111 labeled FnBPA5 in this study, which facilitates research activities thanks to its relatively long half-life of 2.8 days and its good spectral properties, we are aware that for a potential clinical translation a PET-tracer yielding better resolution and accordingly lower radiation exposure would be preferable. Fortunately, indium-111 and gallium-68 possess similar chelating properties, so that NODAGA-FnBPA5 could be easily chelated with gallium-68 instead of indium-111.

To conclude, the development of an imaging agent for the detection of symptomatic endometriosis would be very valuable as it could significantly facilitate the diagnosis, thereby shortening the time-to-diagnosis for the patients and potentially reduce the need for biopsies. In addition, the use of such a tracer would tremendously simplify surgical planning by locating lesions prior to surgery.

## Conclusion

Our investigation employing the preclinical radiotracer [^111^In]In-FnBPA5, specifically targeting relaxed Fn, showed that relaxation of Fn in the uterus is estrous cycle dependent. While there is no uptake during progesterone-dependent phases metestrus and diestrus, radiotracer uptake in estrogen-dependent cycle phases estrus and proestrus is significantly increased. Moving to the tissue level, we herein demonstrated that the observed change in radiotracer uptake is mediated by the muscular component of the uterus—the myometrium. While the abundance of myofibroblasts and Fn is constant irrespective of the estrous cycle stage, the proportion of relaxed Fn is significantly higher during proestrus and estrus in contrast to metestrus and diestrus. An increase in estrogen therefore stimulates relaxation of Fn in the myometrium of the mouse uterus. Transferring these findings to the human situation, we showed in biopsies of endometriosis patients, that the targeting moiety FnBPA5 co-localizes with endometriotic stroma. Our findings therefore demonstrate that relaxed Fn fibrils could be a potential novel target to detect endometriotic lesions and that FnBPA5 could potentially be used as a targeting vector for the noninvasive detection of endometriosis in patients.

## Materials and methods

### General

Peptides equipped with a radiometal chelator or a fluorescent label (Cyanine 5) at the N-terminus were synthesized either in-house or were ordered from Peptide Specialty Laboratories (PSL, Heidelberg, Germany) or piCHEM AG (Grambach, Austria).

### Radiolabeling

FnBPA5 and FnBPA5 scr equipped with a NODAGA-chelator were radiolabeled with [^111^In]InCl_3_ (Mallinckrodt, Cham, Switzerland) in metal-free ammonium acetate (0.5 M, pH 5.5) at a molar activity of 6 MBq/nmol. After 30 min incubation at 50 °C, the solution was subjected to quality control using RP-HPLC (Agilent 1200 Series, Santa Clara, USA) connected to a GinaStar Elysia-Raytest γ-detector (Straubenhardt, Germany). A C18 column (ReproSil-Pur 120, 3 μm, Dr. Maisch GmbH, Germany) was used and eluted with a linear gradient ranging from 95 to 5% of solvent A in 10 min (A: H_2_O containing 0.1% trifluoroacetic acid (TFA); B: acetonitrile). Typically, radiolabeling efficiencies of > 95% were achieved. Typical HPLC chromatograms of [^111^In]In-FnBPA5 and [^111^In]In-FnBPA5 scr are shown in Additional file 1: Figure S1.

### Animal studies

Animal studies were conducted in accordance with the Swiss law on animal protection and procedures were approved by the cantonal authorities of Switzerland under the license number AG75700. Due to the explorative nature of the study, female CD1 mice of reproductive age (9–28 weeks old) included in other experiments within our institute as well as animals specifically allocated to this study were investigated in the biodistribution study. The study included a total of 66 mice, both healthy animals and animals bearing a xenografted tumor, and consequently, treatments were applied at different time-points. For SPECT/CT only healthy CD1 mice were investigated. A clear overview over all animals used, their age and health status is described in Additional file 1: Table S4. Due to the nature of the experiment, no blinding or random allocation to treatment groups was required. Instead the estrous cycle of the mice was continuously followed by vaginal smear analysis in order to obtain enough animals in each cycle stage. However, cycle stage was only analyzed postmortem, so that blinding was given and allocation to the study group as well. Mice were housed in standard cages with a house and nesting material, at room temperature and with a 12 h light–dark cycle. They had access to food and water ad libitum.

### Estrous cycle stage determination

The stage of estrous was determined postmortem via vaginal cytology. For this, the vagina of the mice was flushed with 50 μL of PBS using a pipette. Smears were stained using Crystal violet and according to Papanicolaou before microscopic analysis at × 40 magnification. For the determination of the cycle stage, the estrous cycle tool published by Byers et al. [56] was consulted. Examples of typical images used for estrous cycle identification can be found in the Additional file 1: (Figure S2). The estrous cycle in mice usually lasts 4–5 days and is separated into the following stages: proestrus (P), estrus (E), metestrus (M) and diestrus (D).

### SPECT/CT scans

Mice were subjected to daily vaginal smear analysis in order to detect the adequate time for tracer injection, so that after 24 h mice in each cycle stage would be obtained. SPECT/CT scans were then conducted 24 h after i.v. radiotracer injection of 10–15 MBq (1,7–2,5 nmol) of [^111^In]In-FnBPA5 using a NanoSPECT/CT camera (Mediso Medical Imaging Systems, Budapest, Hungary) in a total of 17 mice. After induction of anesthesia using isoflurane/oxygen, mice were first subjected to a CT scan of 5–10 min and consequently to a SPECT scan of 45–60 min. Image reconstruction and analyses were achieved using HiSPECT (Version 1.4.3049, Scivis GmbH, Göttingen, Germany) and VivoQuant (Version 3.5, inviCRO Imaging Services and Software, Boston USA), respectively. Activity scale was adjusted for maximal contrast and is displayed on each graphic with the corresponding scale in Bq/Voxel. At the end of each scan, mice were subjected to vaginal smear analysis. SPECT/CT scans of all animals in the corresponding cycle stages are provided in Additional file 1: Figure S3.

### Biodistribution

Mice were subjected to daily vaginal smear analysis in order to detect the adequate time for tracer injection, so that after 24 h mice in each cycle stage would be obtained. If timing seemed adequate, 150 kBq (25 pmol) of [^111^In]In-FnBPA5 or 150 kBq (25 pmol) of the control peptide [^111^In]In-FnBPA5 scr in 100 uL of PBS were injected i.v. into the tail vein of a total of 49 mice (29 for [^111^In]In-FnBPA5 and 20 for [^111^In]In-FnBPA5 scr). The number of animals per cycle stage investigated in the biodistribution study is provided in Additional file 1: Table S2 and Table S4. Mice were euthanized 24 h after injection using CO_2_. Consequently, organs were harvested, weighed and radioactivity measured using a gamma counter (Packard Cobra II Auto Gamma, PerkinElmer, Switzerland). Employing 10 μL of the injected solution as a standard, the percentage of the injected activity per gram organ (% iA/g) was calculated.

### Mouse tissue preparation

Uteri from mice subjected to SPECT/CT were euthanized after imaging using CO_2_ and instantly perfused through the left heart ventricle with ice-cold Ringer solution supplemented with 50′000 Units/L heparin. Thereafter, we perfused the animals with a mixture of 1:1 TissueTEK/PBS before the organs of interest were excised and embedded in TissueTEK on dry ice. After complete freezing, the organs were cut into 8–10 μm sections in a cryostat and stored at − 80 °C before further processing.

### Human endometriosis tissue collection and processing

12 patients with suspected endometriosis and undergoing explorative laparoscopy at Kantonsspital Baden (Switzerland) were recruited for the study. No other enrollment criteria were specified. All proceedings were approved by the ethics committee of the Kantonsspital Baden and participants provided informed written consent. Samples were obtained from rapid incision right after surgical removal as frozen, non-fixed sections.

### Immunohistochemistry of mouse and human tissue

Non-fixed mouse and human cryosections were thawed, rehydrated with PBS and blocked with 4% BSA for 30 min. We then incubated the sections with 5 μg/mL Cy5-FnBPA5 or Cy5-FnBPA5 scr for 1 h. After washing, tissues were fixed in 4% paraformaldehyde for 10 min and permeabilized with 0.1% Triton X-100 for 30 min. In order to reduce unspecific binding of antibodies, mouse tissues were blocked using animal-free blocking solution (SP5035, Vector Laboratories, USA), while human tissues were blocked using goat serum (Sigma Aldrich, G9023-10ML) for 1 h. Both sera were supplemented with 0.3 M glycine and 0.01% Triton X-100. Consequently, we incubated the tissue sections with the primary antibody (1° Ab) overnight at 4 °C. Next day, the unbound 1° Ab was washed away and the sections were incubated with the 2° Ab for 1 h, before counter-staining with DAPI (2 μg/mL) for 10 min. The following antibodies were employed: rabbit polyclonal Ab against Fn (Abcam, ab23750), rabbit polyclonal Ab against αSMA (Abcam, ab5694) as 1° antibodies and anti-rabbit polyclonal Ab (Invitrogen, A11034) as 2° antibody as well as rabbit IgG (Vector I-1000) as a control antibody. Slides were imaged using a slide scanner (Pannoramic Midi II, 3D Histech, Hungary), and image analysis was conducted using SlideViewer software (3D, Histech, Hungary) or Fiji [57]. First, unspecific antibody binding as well as background staining was deduced. The myometrium was mapped by hand, and the intensity of Fn, FnBPA5 as well as αSMA was recorded and consequently normalized to the DAPI signal in the same area. The obtained ratio for diestrus was set to 1, and all other values were adjusted accordingly.

### Statistical analysis

Statistical analyses were performed in GraphPad Prism 8. Parametric or nonparametric distribution was assessed using either F test, Bartlett’s or Brown-Forsythe test as provided by GraphPad Prism. Statistical significance of two parametric groups was conducted using unpaired Student’s t test, while two nonparametric groups were analyzed using Mann–Whitney test. Comparisons of three or more equally distributed groups were compared using one-way ANOVA with Dunnet’s post hoc test and nonparametric equivalents were subjected to Kruskal–Wallis test with Dunn’s post hoc test. Statistical significances are given as *p < 0.05. Potential outliers in a dataset were identified using GraphPad Prism’s Outlier Analysis test based on Grubb with *α* = 0.05.

### Supplementary Information

**Additional file 1:** Contains raw data of biodistribution studies, a complete set of SPECT/CT images, images and analysis parameters from fluorescence microscopy as well as supplementary information on HPLC chromatography and animal experiments.

## Data Availability

The data generated for this study are available upon reasonable request from the corresponding author.
