# Supplementary material for: Relaxed fibronectin: a potential novel target for imaging endometriotic lesions
Source: EJNMMI Res. 2024 Feb 10;14:17. doi: 10.1186/s13550-024-01070-0 (PMC10858858; doi:10.1186/s13550-024-01070-0)
Supplement: Supplementary file 1 — Additional file 1: Contains raw data of biodistribution studies, a complete set of SPECT/CT images, images and analysis parameters from fluorescence microscopy as well as supplementary information on HPLC chromatography and animal experiments. [file 13550_2024_1070_MOESM1_ESM.docx]

## Supporting Information

**Relaxed Fibronectin - A Potential Novel Target for Imaging Endometriotic Lesions**

Belinda Trachsel^†‡^, Stefan Imobersteg^†^, Giulia Valpreda^†‡^, Gad Singer†, Regula Grabherr†, Mark Ormos†, Irene A. Burger†, Rahel A. Kubik-Huch†, Roger Schibli^†‡^, Viola Vogel^§^, Martin Béhé^†*^

^†^ Center for Radiopharmaceutical Sciences ETH-PSI-USZ, Paul Scherrer Institute, 5232 Villigen-PSI, Switzerland

^‡^ Department of Chemistry and Applied Biosciences, Institute of Pharmaceutical Sciences, ETH Zurich, 8093 Zurich, Switzerland

^§^ Laboratory of Applied Mechanobiology, Institute of Translational Medicine, Department of Health Sciences and Technology, ETH Zurich, 8093 Zurich, Switzerland

†Kantonsspital Baden, 5404 Baden, Switzerland

* Corresponding author email address: [martin.behe@psi.ch](mailto:martin.behe@psi.ch), Forschungsstrasse 111, 5232 Villigen, Switzerland

### Radiolabelling


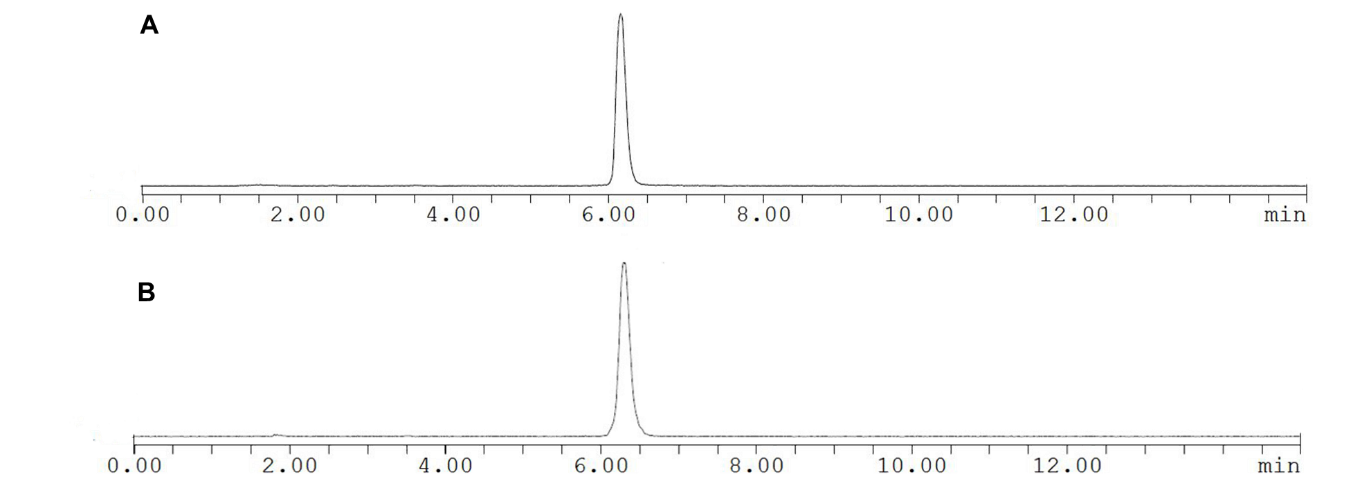


***Figure S1.*** *HPLC radiochromatograms of* ***(a)*** *[^111^In]In-FnBPA5 and* ***(b)*** *[^111^In]In-FnBA5 scr.*

### Biodistribution Data

***Table S1.*** *Mean organ uptake in percentage of injected activity per gram (% iA/g) and standard deviation expressed in absolute values or as percentage of the mean 24 h after injection of [111In]In-FnBPA5 into female CD1 mice. Of special interest is the large standard deviation of the uterus, which is equivalent to 87 % of the mean % iA/g.*

| Organ | % iA/g | SD | SD [%] |
| --- | --- | --- | --- |
| Blood | 0.25 | 0.078 | 31 |
| Muscle | 0.32 | 0.27 | 84 |
| Kidney | 73 | 32 | 44 |
| Liver | 3.3 | 1.7 | 52 |
| Uterus | **5.3** | **4.7** | **87** |
| Ovaries | 2.5 | 1.1 | 44 |

***Figure S2.*** *The complete set of biodistribution data of mice in different estrous cycle stages at 24 h p.i. Statistical analysis was conducted using multiple t-test with Dunn-Bonferroni multiple comparison analysis in GraphPad Prism. The corresponding data can be found in table S8.*

***Table S2.*** *Biodistribution data of [^111^In]In-FnBPA5 and the control [^111^In]In-FnBPA5 scr depending on the cycle stage of the mouse. Data is given as the mean percentage of injected activity per gram (% iA/g) organ ± standard deviation.*

|  | **Proestrus** | | **Estrus** | |
| --- | --- | --- | --- | --- |
|  | **[^111^In]-FnBPA5**  **(n = 5)** | **[^111^In]-FnBPA5 scr**  **(n = 3)** | **[^111^In]-FnBPA5**  **(n = 4)** | **[^111^In]-FnBPA5 scr**  **(n = 2)** |
| Blood | 0.31 ± 0.09 | 0.04 ± 0.02 | 0.21 ± 0.02 | 0.04 ± 0.01 |
| Heart | 0.66 ± 0.31 | 0.06 ± 0.02 | 0.46 ± 0.09 | 0.04 ± 0.00 |
| Lung | 1.27 ± 0.60 | 0.06 ± 0.04 | 0.89 ± 0.16 | 0.04 ± 0.00 |
| Spleen | 3.34 ± 1.27 | 0.08 ± 0.04 | 2.45 ± 0.64 | 0.06 ± 0.00 |
| Liver | 4.76 ± 2.49 | 0.09 ± 0.04 | 3.26 ±0.69 | 0.06 ± 0.00 |
| Intestines | 1.35 ± 0.51 | 0.04 ± 0.03 | 1.02 ± 0.16 | 0.03 ± 0.01 |
| Uterus | 8.67 ± 5.42 | 0.14 ± 0.09 | 10.38 ± 4.98 | 0.08 ± 0.02 |
| Ovaries | 2.90 ± 0.82 | 0.31 ± 0.15 | 3.24 ± 0.73 | 0.15 ± 0.06 |
| Muscle | 0.52 ± 0.47 | 0.03 ± 0.01 | 0.25 ± 0.03 | 0.02 ± 0.01 |
| Bone | 1.37 ± 0.67 | 0.23 ± 0.21 | 1.19 ± 0.17 | 0.13 ± 0.02 |
| Kidney | 84.29 ± 31.86 | 143.29 ± 56.97 | 94.11 ± 12.84 | 86.89 ± 11.48 |

|  | **Metestrus** | | **Diestrus** | |
| --- | --- | --- | --- | --- |
|  | **[^111^In]-FnBPA5**  **(n = 7)** | **[^111^In]-FnBPA5 scr**  **(n = 5)** | **[^111^In]-FnBPA5**  **(n = 13)** | **[^111^In]-FnBPA5 scr**  **(n = 10)** |
| Blood | 0.24 ± 0.08 | 0.05 ± 0.01 | 0.33 ± 0.21 | 0.05 ± 0.03 |
| Heart | 0.45 ± 0.23 | 0.05 ± 0.02 | 0.57 ± 0.32 | 0.05 ± 0.02 |
| Lung | 0.87 ± 0.50 | 0.06 ± 0.02 | 1.07 ± 0.58 | 0.06 ± 0.02 |
| Spleen | 2.06 ± 0.93 | 0.08 ± 0.03 | 3.54 ± 2.58 | 0.07 ± 0.03 |
| Liver | 2.73 ± 1.52 | 0.08 ± 0.02 | 5.33 ± 4.12 | 0.07 ± 0.03 |
| Intestines | 0.92 ± 0.40 | 0.12 ± 0.02 | 1.11 ± 0.49 | 0.04 ± 0.02 |
| Uterus | 2.56 ± 1.44 | 0.24 ± 0.04 | 2.68 ± 1.35 | 0.35 ± 0.74 |
| Ovaries | 2.09 ± 1.15 | 0.24 ± 0.04 | 2.75 ± 1.97 | 0.22 ± 0.08 |
| Muscle | 0.26 ± 0.20 | 0.04 ± 0.04 | 0.37 ± 0.29 | 0.08 ± 0.14 |
| Bone | 1.02 ± 0.84 | 0.20 ± 0.07 | 1.54 ± 1.08 | 0.23 ± 0.20 |
| Kidney | 62.91 ± 34.72 | 140.32 ± 44.78 | 93.33 ± 44.96 | 130.44 ± 42.64 |

### Depiction of the Estrous Cycle in the Mouse


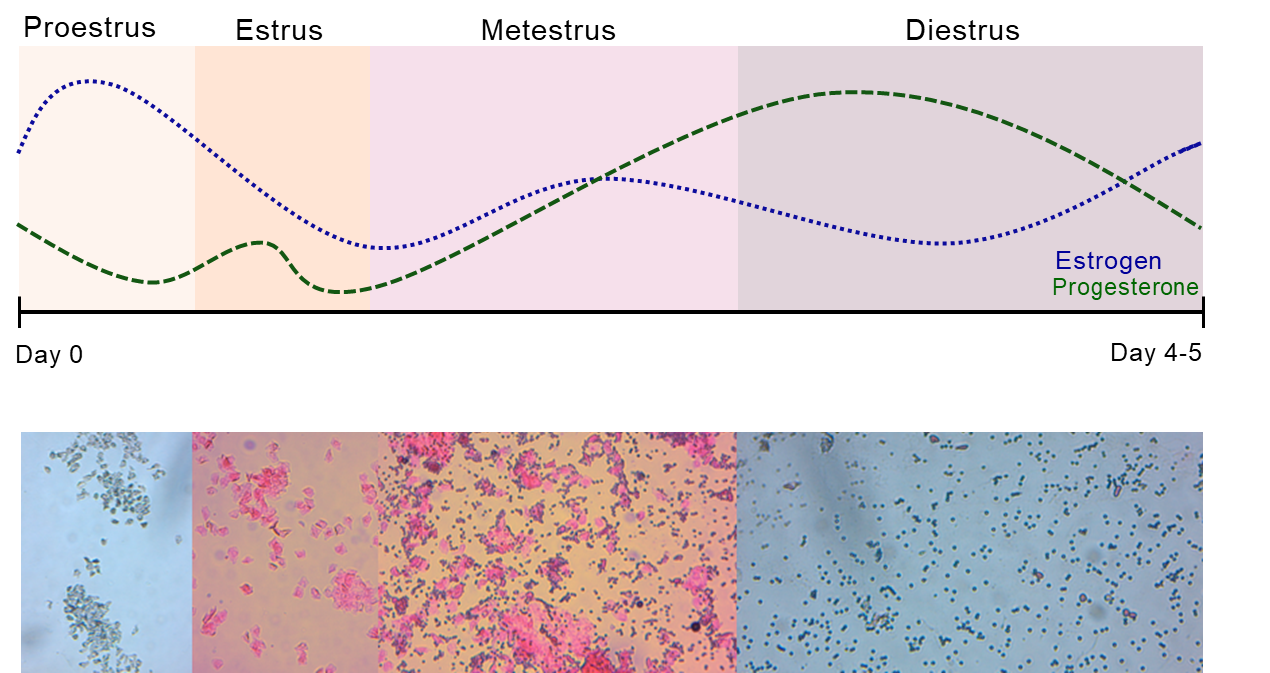


***Figure S2.*** *Top:* *Scheme of the estrous cycle in the mouse. The estrous cycle in the mouse usually lasts between four and five days and is separated into four different cycle stages: Proestrus (P), Estrus (E), Metestrus (M) and Diestrus (D). Depicted in blue and green are the relative abundances of estrogen and progesterone along the estrous cycle. Graphic adapted from schemes published by Hong and Choi (58) and from Wang and Dey (59). Bottom: The estrous cycle stage of an individual mouse can easily be characterized by analysing the cellular composition of a vaginal smear. Shown here are examples from our lab of how smears in different cycle stages look like under the microscope after staining according to Papanicolaou.*

###
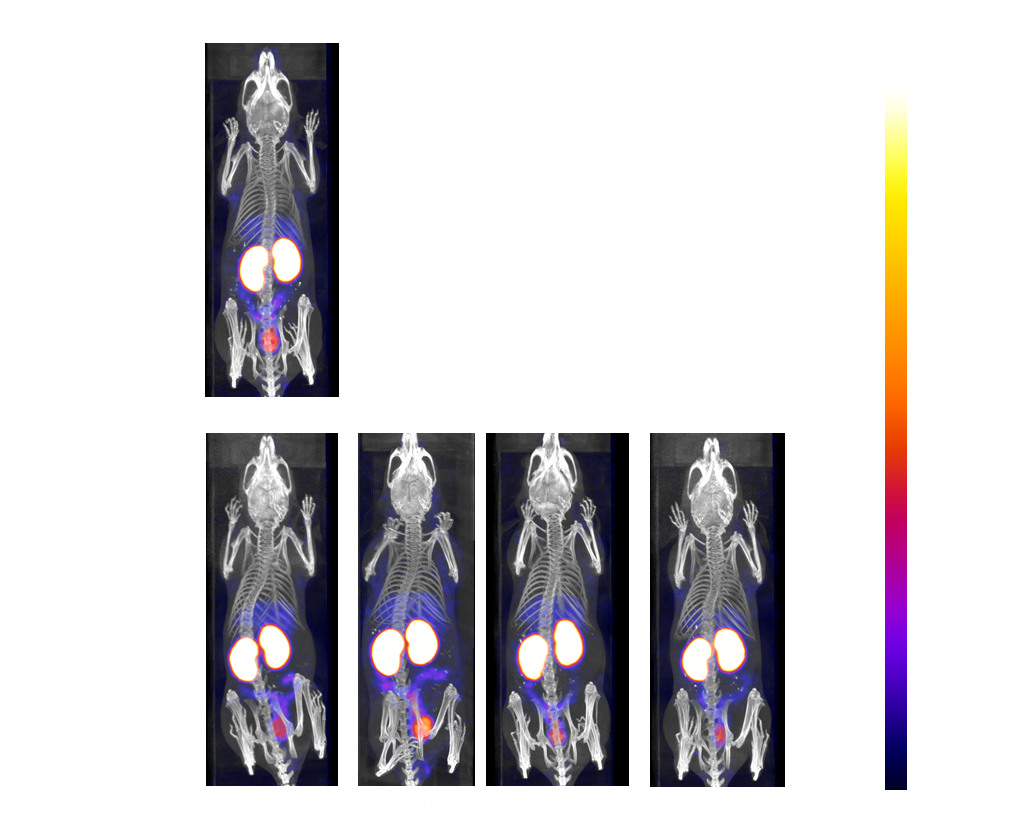
SPECT/CT Images

**
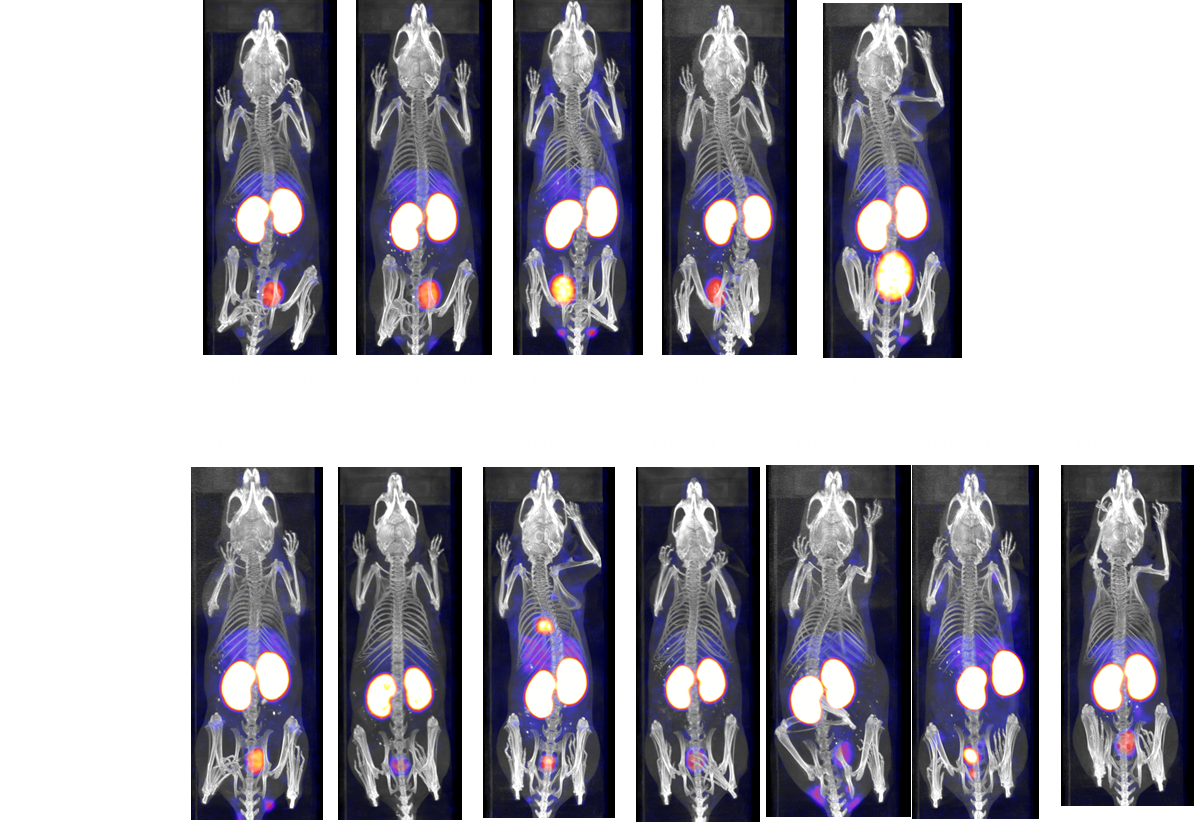
**

***Figure S3.*** *SPECT/CT scans of female CD1 mice injected with [^111^In]In-FnBPA5 24 h p.i. grouped by the respective estrous cycle stage. All scans were adjusted to a scale ranging from 0 to 8 Bq/Voxel. While during proestrus and estrus animals display a signal in the uterus, there is no such signal visible in mice during metestrus and diestrus.*

### Fluorescence Images from Mouse Uteri and Their Quantification


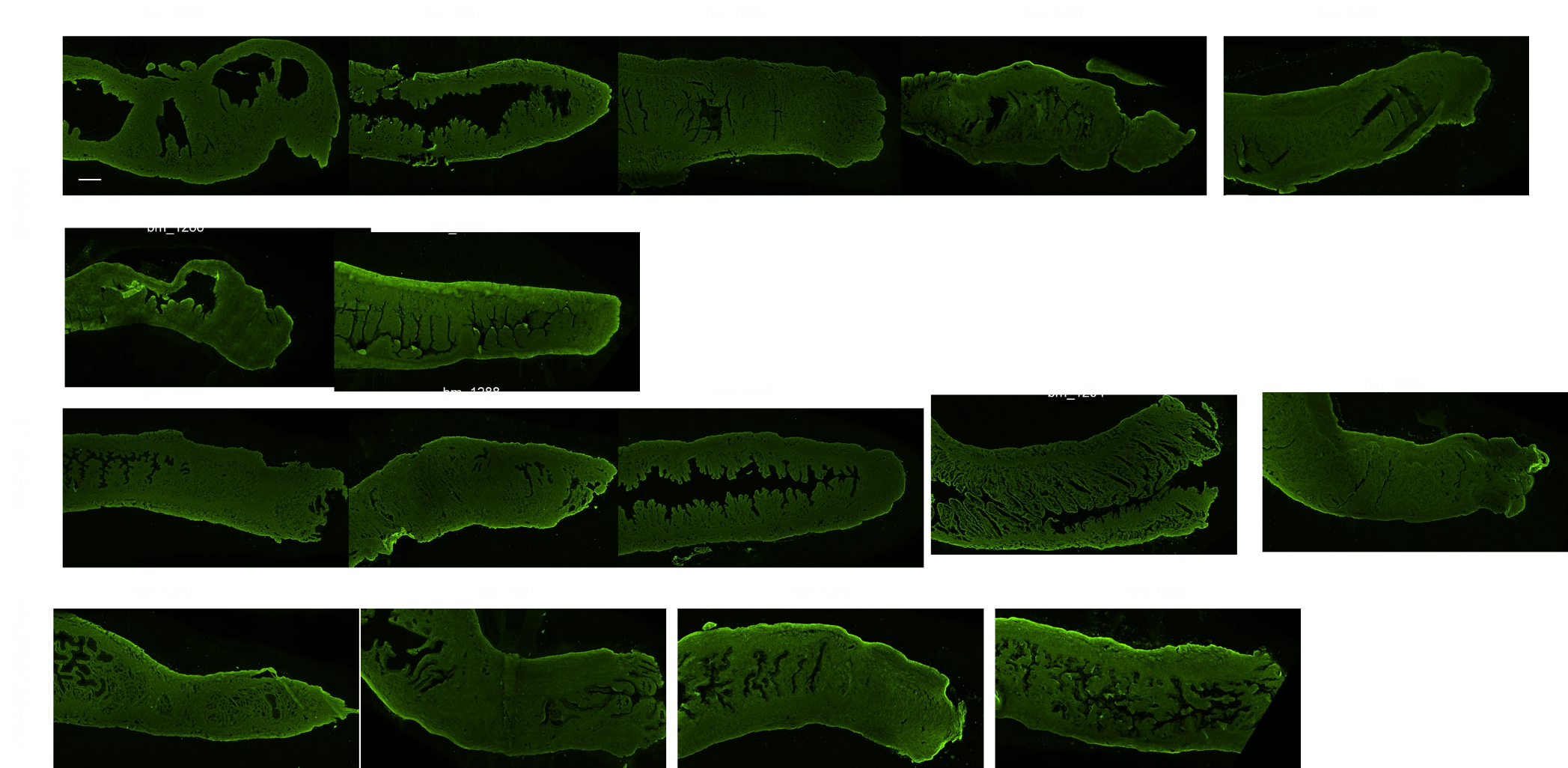


***Figure S4a.*** *Images of mouse uteri stained using IHC. Fixed mouse uteri were stained with a polyclonal antibody against Fn and Fn content in the myometrium was quantified and normalized to the DAPI signal to obtain quantitative data (see Table S9). Images were adjusted to a white scale from 0 to 150 to allow better visualization of the Fn signal. Note, that for quantification channels were not adjusted and areas containing artefacts were omitted. Scale bar = 500 μm.*

***
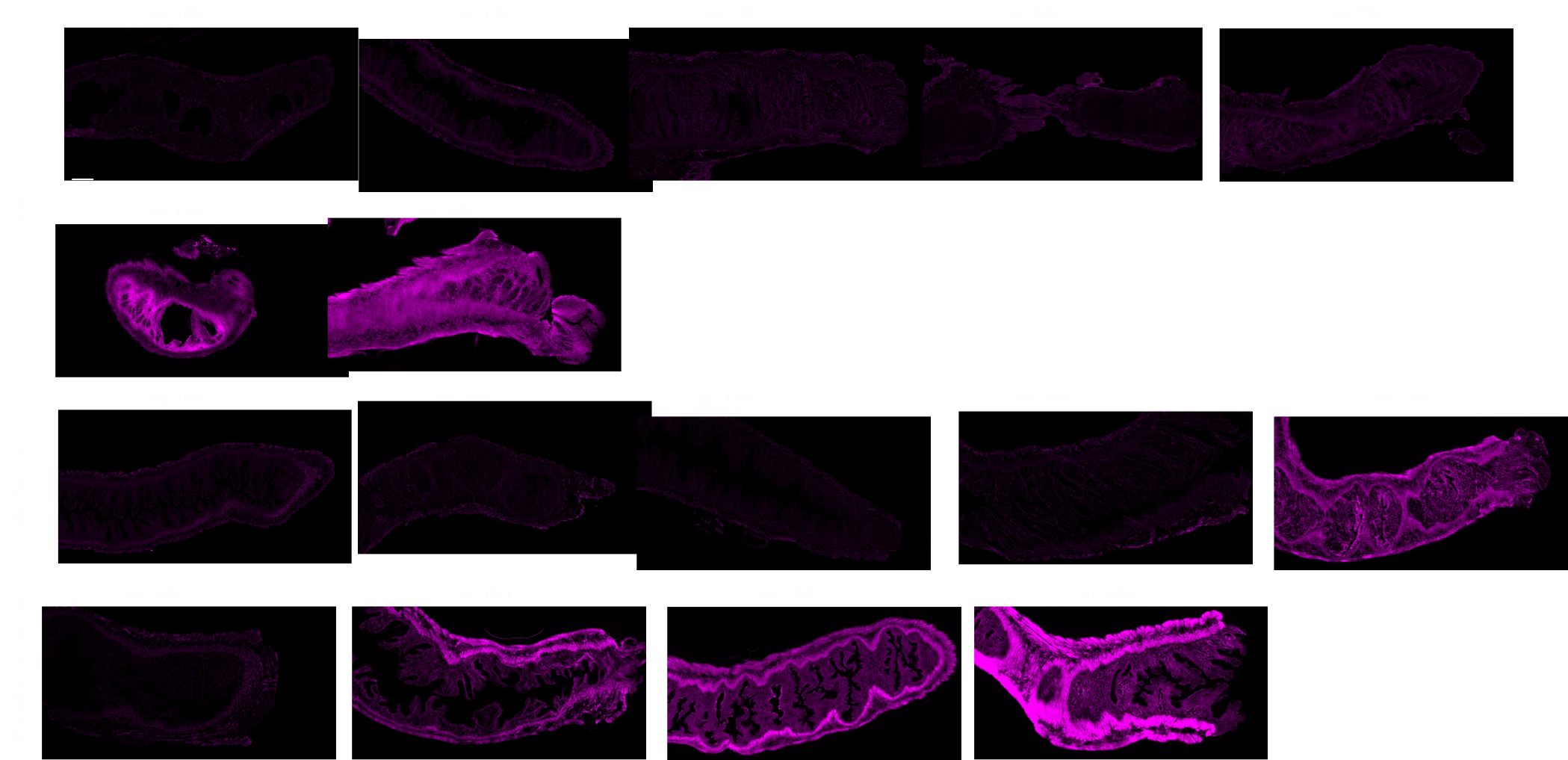
***

***Figure S4b.*** *Images of mouse uteri stained using IHC. Fixed mouse uteri were stained with Cy5-FnBPA5 and relaxed Fn content in the myometrium was quantified and normalized to the DAPI signal to obtain quantitative data (see Table S9). Images were adjusted to a white scale from 0 to 200 to allow better visualization of the Cy5-FnBPA5 signal. Note, that for quantification channels were not adjusted and areas containing artefacts were omitted. Scale bar = 500 μm****.***

***
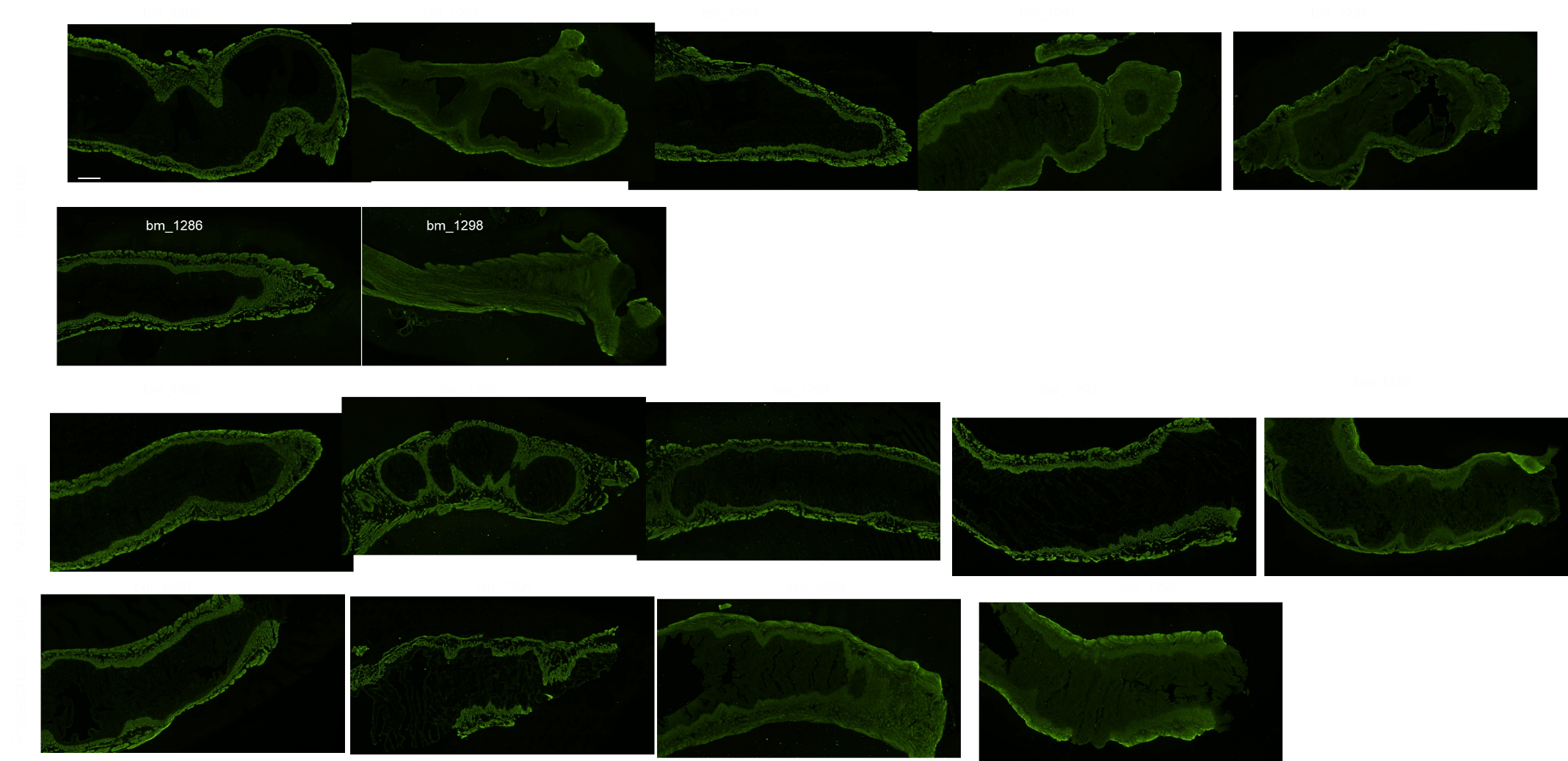
***

***Figure S4c.*** *Images of mouse uteri stained using IHC. Fixed mouse uteri were stained with an antibody against αSMA and αSMA expression in the myometrium was quantified and normalized to the DAPI signal to obtain quantitative data (see Table S9). Images were adjusted to a white scale from 0 to 150 to allow better visualization of the green αSMA signal. Note, that for quantification channels were not adjusted and areas containing artefacts were omitted. Scale bar = 500 μm****.***

***Table S3.*** *Raw data from quantification of fluorescence signal in the myometrium. The sample ID corresponds to the same animals employed in the SPECT/CT study. The signal of αSMA, Fn and Cy5-FnBPA5 was normalized to the DAPI signal of the same region. These values were later transferred to fold changes by setting the average ratio of diestrus equal to 1.*

| **Sample ID** | **Cycle Stage** | **αSMA / DAPI** | **Fn/ / DAPI** | **Cy5-FnBPA5 / DAPI** |
| --- | --- | --- | --- | --- |
| bm_1289 | Metestrus | 0.5 | 3.4 | 4.8 |
| bm_1297 | Diestrus | 0.7 | 3.8 | 1.4 |
| bm_1295 | Metestrus | 0.7 | 7.9 | 1.4 |
| bm_1298 | Diestrus | 0.3 | 1.4 | 3.8 |
| bm_1291 | Diestrus | 0.4 | 0.3 | 0.9 |
| bm_1290 | Estrus | 0.5 | 3.0 | 2.2 |
| bm_1294 | Metestrus | 0.7 | 4.1 | 1.5 |
| bm_1292 | Metestrus | 0.4 | 3.4 | 1.1 |
| bm_1303 | Diestrus | 0.8 | 3.3 | 1.1 |
| bm_1288 | Metestrus | 0.6 | 4.5 | 1.6 |
| bm_1296 | Estrus | 0.5 | 1.6 | 7.5 |
| bm_1299 | Estrus | 0.4 | 2.1 | 6.7 |
| bm_1293 | Diestrus | 0.8 | 4.9 | 1.2 |
| bm_1286 | Diestrus | 0.4 | 4.4 | N/A |
| bm_1287 | Diestrus | 0.3 | 1.6 | 1.4 |
| bm_1301 | Proestrus / Estrus | 0.8 | 2.0 | 5.4 |

N/A: no quantification due to bad image quality

### Human Endometriosis Samples


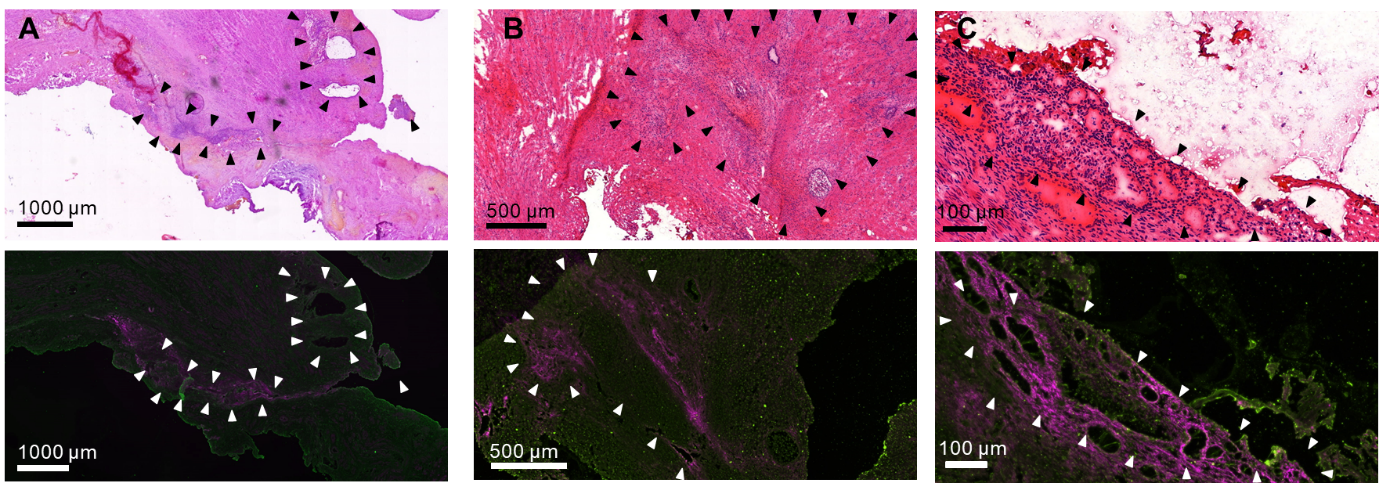


***Figure S5a.*** *HE (top) and IHC (bottom) staining of human biopsies with suspected endometriosis. IHC images were stained for Fn (green) with a polyclonal antibody and for relaxed Fn with Cy5-labelled FnBPA5 (pink). Arrows indicate areas containing endometriotic stroma and/or epithelium.* ***(A)*** *Extensive areas of endometriosis including cyst formation. FnBPA5 stains strongly in one area, while it stains only faintly around the endometriotic cysts.* ***(B)*** *This biopsy displays a large amount of endometriosis containing stroma, epithelium and cysts. FnBPA5 stains strongly in areas of endometriotic stroma, but does not stain the endometriotic epithelium, which surrounds the cysts.*

*
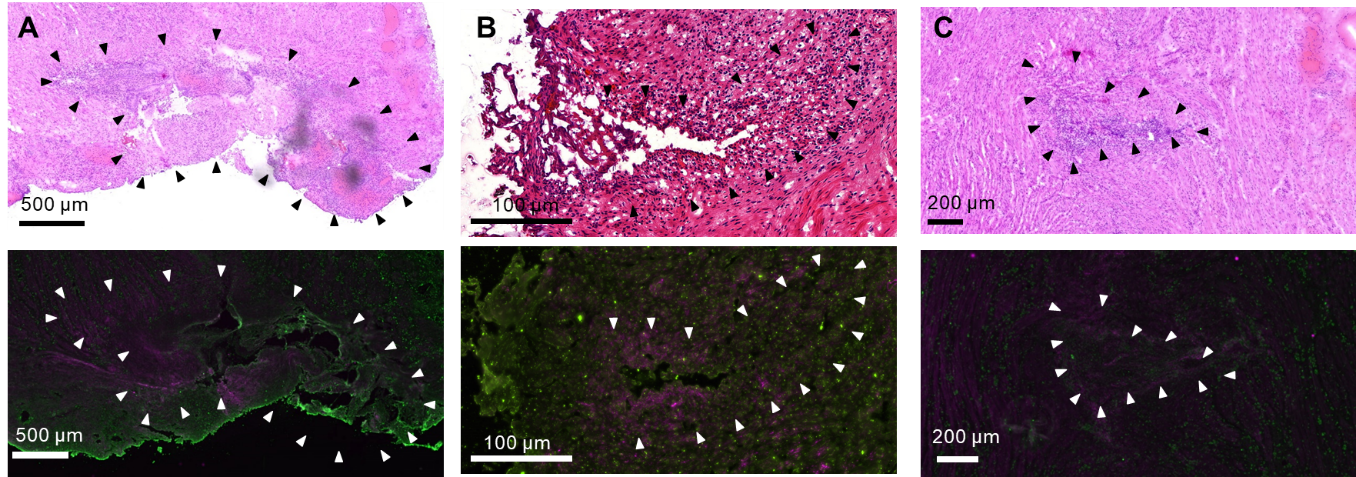
*

***Figure S5b.*** *HE (top) and IHC (bottom) staining of human biopsies with suspected endometriosis. IHC images were stained for Fn (green) with a polyclonal antibody and for relaxed Fn with Cy5-labelled FnBPA5 (pink). Arrows indicate areas containing endometriotic stroma and/or epithelium.* ***(A)*** *This biopsy displays an area with both endometriotic stroma and epithelium. Cy5-FnBPA5 stains strongly.* ***(B)*** *Area containing an endometriotic gland lined by endometriotic epithelium. There is Cy5-FnBPA5 signal in the vicinity of the endometriotic gland.* ***(C)*** *Endometriotic stroma with few epithelial components. There is faint Cy5-FnBPA5 signal surrounding the endometriotic region and on smooth muscle fibers in the vicinity.*

**Description of Experimental Animals**

***Table S4.*** *An overview of the animals included in the biodistribution study, their attribution to either the control group ([^111^In]In-FnBPA5 scr) or the treatment group ([^111^In]In-FnBPA5), time-point of experiment, age of animals, the estrous cycle stage at time of necropsy as well as the % iA/g uterus.*

| **Mouse Identifier** | **Group** | **Treatment Period** | **Age** | **Health Status** | **Cycle Stage** | **% iA/g Uterus** |
| --- | --- | --- | --- | --- | --- | --- |
| C1 | BioD _Control | August 2022 | 9-10 w | Healthy | Metestrus | 0.12 |
| C2 | BioD _Control | August 2022 | 9-10 w | Healthy | Diestrus | 0.07 |
| C3 | BioD _Control | August 2022 | 9-10 w | Healthy | Diestrus | 2.44 |
| C4 | BioD _Control | August 2022 | 9-10 w | Healthy | Diestrus | 0.08 |
| C5 | BioD _Control | August 2022 | 9-10 w | Healthy | Diestrus | 0.06 |
| C6 | BioD _Control | August 2022 | 9-10 w | Healthy | Metestrus | 0.09 |
| C7 | BioD _Control | August 2022 | 9-10 w | Healthy | Diestrus | 0.07 |
| C8 | BioD _Control | August 2022 | 9-10 w | Healthy | Diestrus | 0.1 |
| C9 | BioD _Control | August 2022 | 9-10 w | Healthy | Metestrus | 0.12 |
| C10 | BioD _Control | August 2022 | 9-10 w | Healthy | Diestrus | 0.27 |
| C11 | BioD _Control | August 2022 | 9-10 w | Healthy | Diestrus | 0.17 |
| C12 | BioD _Control | August 2022 | 9-10 w | Healthy | Metestrus | 0.13 |
| C13 | BioD _Control | August 2022 | 9-10 w | Healthy | Metestrus | 0.16 |
| C14 | BioD _Control | August 2022 | 9-10 w | Healthy | Proestrus | 0.24 |
| C15 | BioD _Control | August 2022 | 9-10 w | Healthy | Proestrus | 0.11 |
| C16 | BioD _Control | August 2022 | 9-10 w | Healthy | Proestrus | 0.06 |
| C17 | BioD _Control | August 2022 | 9-10 w | Healthy | Diestrus | 0.14 |
| C18 | BioD _Control | August 2022 | 9-10 w | Healthy | Diestrus | 0.12 |
| C19 | BioD _Control | August 2022 | 9-10 w | Healthy | Estrus | 0.1 |
| C20 | BioD _Control | August 2022 | 9-10 w | Healthy | Estrus | 0.07 |
| T1 | BioD _FnBPA5 | March 2021 | 9 w | 67NR breast tumour^1^ | Diestrus | 4.11 |
| T2 | BioD _FnBPA5 | March 2021 | 9 w | 67NR breast tumour^1^ | Proestrus | 17 |
| T3 | BioD _FnBPA5 | March 2021 | 9 w | 67NR breast tumour^1^ | Metestrus | 2.42 |
| T4 | BioD _FnBPA5 | March 2021 | 9 w | 67NR breast tumour^1^ | Diestrus | 1.77 |
| T5 | BioD _FnBPA5 | April 2021 | 10 w | PC-3 sc tumour shoulders ^2^ | Metestrus | 4.72 |
| T6 | BioD _FnBPA5 | April 2021 | 10 w | PC-3 sc tumour ^2^shoulders ^2^ | Diestrus | 2.08 |
| T7 | BioD _FnBPA5 | April 2021 | 10 w | PC-3 sc tumour shoulders ^2^ | Diestrus | 1.79 |
| T8 | BioD _FnBPA5 | October 2021 | 13 w | Hormone Induction^3^ | Estrus | 8.87 |
| T9 | BioD _FnBPA5 | October 2021 | 13 w | Hormone Induction^3^ | Diestrus | 1.4 |
| T10 | BioD _FnBPA5 | October 2021 | 13 w | Hormone Induction^3^ | Diestrus | 2.2 |
| T11 | BioD _FnBPA5 | December 2021 | 28 w | Healthy | Proestrus | 6.778 |
| T12 | BioD _FnBPA5 | December 2021 | 28 w | Healthy | Proestrus | 6.45 |
| T13 | BioD _FnBPA5 | December 2021 | 28 w | Healthy | Metestrus | 1.67 |
| T14 | BioD _FnBPA5 | December 2021 | 28 w | Healthy | Metestrus | 2.39 |
| T15 | BioD _FnBPA5 | February 2022 | 9-10 w | Healthy | Proestrus | 10.36 |
| T16 | BioD _FnBPA5 | February 2022 | 9-10 w | Healthy | Proestrus | 2.69 |
| T17 | BioD _FnBPA5 | February 2022 | 9-10 w | Healthy | Estrus | 4.7 |
| T18 | BioD _FnBPA5 | February 2022 | 9-10 w | Healthy | Estrus | 11.3 |
| T19 | BioD _FnBPA5 | February 2022 | 9-10 w | Healthy | Estrus | 16.64 |
| T20 | BioD _FnBPA5 | February 2022 | 9-10 w | Healthy | Metestrus | 4.3 |
| T21 | BioD _FnBPA5 | February 2022 | 9-10 w | Healthy | Metestrus | 1.73 |
| T22 | BioD _FnBPA5 | February 2022 | 9-10 w | Healthy | Metestrus | 0.73 |
| T23 | BioD _FnBPA5 | February 2022 | 9-10 w | Healthy | Diestrus | 1.8 |
| T24 | BioD _FnBPA5 | February 2022 | 9-10 w | Healthy | Diestrus | 2.08 |
| T25 | BioD _FnBPA5 | February 2022 | 9-10 w | Healthy | Diestrus | 2.05 |
| T26 | BioD _FnBPA5 | February 2022 | 9-10 w | Healthy | Diestrus | 1.58 |
| T27 | BioD _FnBPA5 | February 2022 | 9-10 w | Healthy | Diestrus | 5.76 |
| T28 | BioD _FnBPA5 | February 2022 | 9-10 w | Healthy | Diestrus | 3.8 |
| T29 | BioD _FnBPA5 | February 2022 | 9-10 w | Healthy | Diestrus | 4.27 |
| bm_1289 | SPECT_FnBPA5 | November 2021 | 8-10 w | Healthy | Metestrus | SPECT |
| bm_1297 | SPECT_FnBPA5 | November 2021 | 8-10 w | Healthy | Diestrus | SPECT |
| bm_1295 | SPECT_FnBPA5 | November 2021 | 8-10 w | Healthy | Metestrus | SPECT |
| bm_1298 | SPECT_FnBPA5 | November 2021 | 8-10 w | Healthy | Diestrus | SPECT |
| bm_1291 | SPECT_FnBPA5 | November 2021 | 8-10 w | Healthy | Diestrus | SPECT |
| bm_1290 | SPECT_FnBPA5 | November 2021 | 8-10 w | Healthy | Estrus | SPECT |
| bm_1294 | SPECT_FnBPA5 | November 2021 | 8-10 w | Healthy | Metestrus | SPECT |
| bm_1292 | SPECT_FnBPA5 | November 2021 | 8-10 w | Healthy | Metestrus | SPECT |
| bm_1303 | SPECT_FnBPA5 | November 2021 | 8-10 w | Healthy | Diestrus | SPECT |
| bm_1288 | SPECT_FnBPA5 | November 2021 | 8-10 w | Healthy | Metestrus | SPECT |
| bm_1296 | SPECT_FnBPA5 | November 2021 | 8-10 w | Healthy | Estrus | SPECT |
| bm_1299 | SPECT_FnBPA5 | November 2021 | 8-10 w | Healthy | Estrus | SPECT |
| bm_1293 | SPECT_FnBPA5 | November 2021 | 8-10 w | Healthy | Diestrus | SPECT |
| bm_1286 | SPECT_FnBPA5 | November 2021 | 8-10 w | Healthy | Diestrus | SPECT |
| bm_1287 | SPECT_FnBPA5 | November 2021 | 8-10 w | Healthy | Diestrus | SPECT |
| bm_1301 | SPECT_FnBPA5 | November 2021 | 8-10 w | Healthy | Proestrus/Estrus | SPECT |

**^1^** Mice were inoculated orthotopically into the mammary fat pad of the 4^th^ inguinal nipples with 40’000 cells of the murine breast cancer cell line 67NR (a kind gift from the lab of Prof. Curzio Rüegg at University of Fribourg, Switzerland) in 50 μL PBS. 15 days after inoculation, mice were injected with [^111^In]In-FnBPA5 and further treated according to the section Biodistribution under Materials & Methods.

**^2^** Mice were inoculated subcutaneously into the shoulder with 5 Mio PC-3 cells (ATCC) in 100 μL PBS. 5 weeks after inoculation, mice were injected with [^111^In]In-FnBPA5 and further treated according to the section Biodistribution under Materials & Methods.

**^3^** In an attempt to induce the estrous cycle stage of the mice, mice were subcutaneously injected with 10 μg/100 g bw estradiol benzoate 48 before radiotracer application and 4-6 h before application with 0.5 mg/100 g bw progesterone. Thereafter mice wer injected with [^111^In]In-FnBPA5 and further treated according to the section Biodistribution under Materials & Methods.
